# Gastrodin alleviates glucocorticoid induced osteoporosis in rats via activating the Nrf2 signaling pathways

**DOI:** 10.18632/oncotarget.23936

**Published:** 2018-01-03

**Authors:** Shengye Liu, Long Zhou, Liyu Yang, Shuai Mu, Tao Fang, Qin Fu

**Affiliations:** ^1^ Department of Spine and Joint Surgery, Shengjing Hospital of China Medical University, Shenyang, Liaoning 110004, P.R. China

**Keywords:** dexamethasone, gastrodin, glucocorticoid induced osteoporosis, reactive oxygen species, Nrf2

## Abstract

**Background:**

Prolonged and over-dosed administration of glucocorticoids results in more bone remodeling, leading to glucocorticoid-induced osteoporosis, which is primarily due to dysfunction and apoptosis of osteoblasts. The present study investigated the therapeutic effect and molecular mechanism of gastrodin, a natural bioactive compound isolated from the traditional Chinese herbal agent Gastrodia elata, on osteoblastic cells *in vivo* and *in vitro*.

**Materials and Methods:**

The anti-dexamethasone (DEX) effects of gastrodin on primary osteoblasts were measured by cell viability, flow cytometry, and western blot analysis *in vitro*, and also extensively examined in a rat model *in vivo*.

**Results:**

The results show that gastrodin pretreatment significantly increased osteoblast viability and alkaline phosphatase activity when exposed to DEX. Alizarin Red staining indicated more calcium deposits formed in the gastrodin pretreatment against DEX group. Gastrodin alleviated DEX-induced reactive oxygen species at both the mitochondrial and cellular levels in osteoblasts. In addition, gastrodin protected primary osteoblasts from caspase3-related apoptosis by reducing the loss in the mitochondrial membrane potential and decreasing the release of DEX-induced cytochrome-C, bax, and apoptosis inducing factor. Gastrodin also antagonized upregulated endoplasmic reticulum stress signals induced by DEX, including the expression of GRP78, CHOP, and phosphorylated eIF2α. Furthermore, gastrodin protected osteoblasts by activating the nuclear factor erythroid derived 2-related factor-2 (Nrf2) pathway. Furthermore, femoral micro-computed tomography scans and biomechanical tests revealed that gastrodin improved bone microstructure and mitigated DEX-induced deterioration in bone quality.

**Conclusions:**

These findings suggest that gastrodin alleviated glucocorticoid-induced osteoporosis in rats by protecting osteoblasts via the Nrf2 regulated mitochondrial and ER stress-related signaling pathways.

## INTRODUCTION

Glucocorticoids (GCs) are widely applied to treat inflammatory and immune-mediated clinical complications. However, glucocorticoid-induced osteoporosis (GIO) has been universally acknowledged as the most common secondary and iatrogenic form of osteoporosis. Over-doses of GCs result in frequent bone loss, which is primarily due to dysfunction and apoptosis of osteoblasts. To elucidate the complex interplay between GCs and altered bone homeostasis is of vital clinical significance.

**Figure 1:**
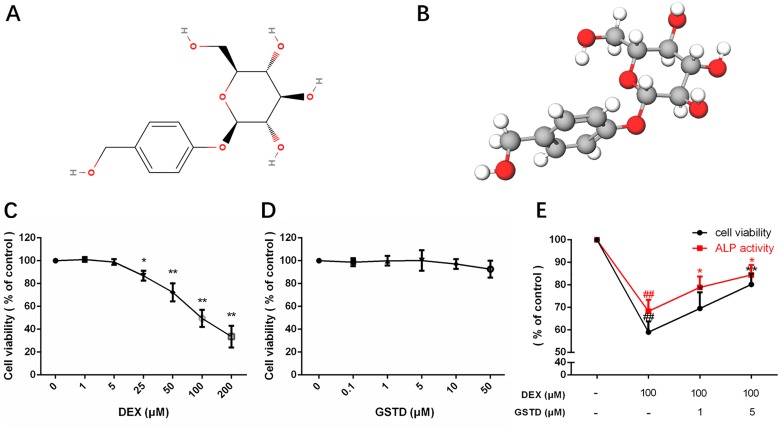
Molecular view of gastrodin and cell viability, ALP activity of primary osteoblasts in drug treatments Structural formula (**A**) and model (**B**). Cell viability and ALP activity of primary osteoblasts under GSTD or DEX treatment were determined by the CCK-8 assay and ALP assay respectively. The measurement was triplicated and data are presented as the mean ± SD. ^##^*P* < 0.01 vs control; ^**^P < 0.01; ^*^P < 0.05 vs DEX100 (*n* = 6).

Gastrodin (GSTD), isolated from the traditional Chinese herbal agent Gastrodia elata, has been confirmed as one of the major active constituents of rhizoma gastrodiae (Figure 1). GSTD has anti-necrosis and anti-aging properties as well as anti-apoptotic activities [1, 2]. It effectively removes oxygen free radicals, enhances antioxidant activity, restrains coupling of oxidative phosphorylation, and increases antioxidant enzymes, such as malondialdehyde and superoxide dismutase. However, its effect on GC-induced dysfunction of primary osteoblasts has not been accurately elucidated.

Mitochondria and the endoplasmic reticulum (ER) are two organelles in cells that participate in apoptotic regulation of osteoblasts. Most studies have suggested that abundant intracellular reactive oxygen species (ROS) production is caused by mitochondria in which the respiratory chain catalyzes the generation of ATP among five integrated complexes [3, 4]. A previous study showed that excessive ER stress and mitochondrial dysfunction are involved in GC-induced apoptosis of osteoblasts and osteocytes [5]. Other studies have revealed that GCs initiate the generation of ROS by different means [6]. Furthermore, ROS contribute to various pathological conditions that drive irreversible destruction of cellular components, including DNA, organelles, and other cytokines, resulting in cell apoptosis or necrosis. Earlier studies demonstrated that ROS-induced ER stress led to apoptosis of osteoblasts in bone as well as decreased mineral deposition *in vitro* [7–9], which was also demonstrated in a model of osteogenesis imperfecta [10]. The significant role of ROS-related apoptosis by mitochondrial dysfunction and ER stress during cell survival requires a detailed investigation of the molecular mechanism and specific determinants of ROS production.

More recent evidence has suggested that nuclear factor erythroid derived 2-related factor-2 (Nrf2) maintains cellular redox homeostasis in bone [11]. Nrf2 initiates the expression of antioxidant enzymes, including NAD (P) H: quinone oxidoreductase 1 (NQO-1) and heme oxygenase 1 (HO-1) [12]. These pivotal molecules are regarded as beneficial factors for improving bone quality in GIO. In addition, GSTD has been demonstrated to be a potential activator of Nrf2 [13]. Taken together, we chose dexamethasone (DEX) as the most common type of GC to show whether the Nrf2 pathway is instrumental in alleviating mitochondrial dysfunction and ER stress by GSTD to maintain normal bone mass in a GIO model.

## RESULTS

### Effects of GSTD on DEXinduced cytotoxicity and alkaline phosphatase (ALP) activity in osteoblasts

As shown in Figure 1C, exposure to DEX significantly suppressed cell viability (> 40% at 100 μM), whereas the DEXinduced reduction in cell viability was prevented by pretreatment with GSTD. GSTD concentrations that did not have any measurable adverse effects on the cells in Figure 1D were selected. To investigate the protective effects on primary osteoblasts, cells were treated with 1 and 5 μM GSTD for 2 h followed by exposure to DEX for 24 h. The results showed that GSTD significantly reduced high-dosed DEX toxicity of osteoblasts in a dose-dependent manner (Figure 1E). ALP is a well-recognized indicator of osteoblast differentiation ability [14, 15]. GSTD rescued ALP activity against DEX (Figure 1E).

### GSTD alleviates DEX-induced mitochondrial membrane permeabilization (MMP) loss in osteoblasts

**Figure 2:**
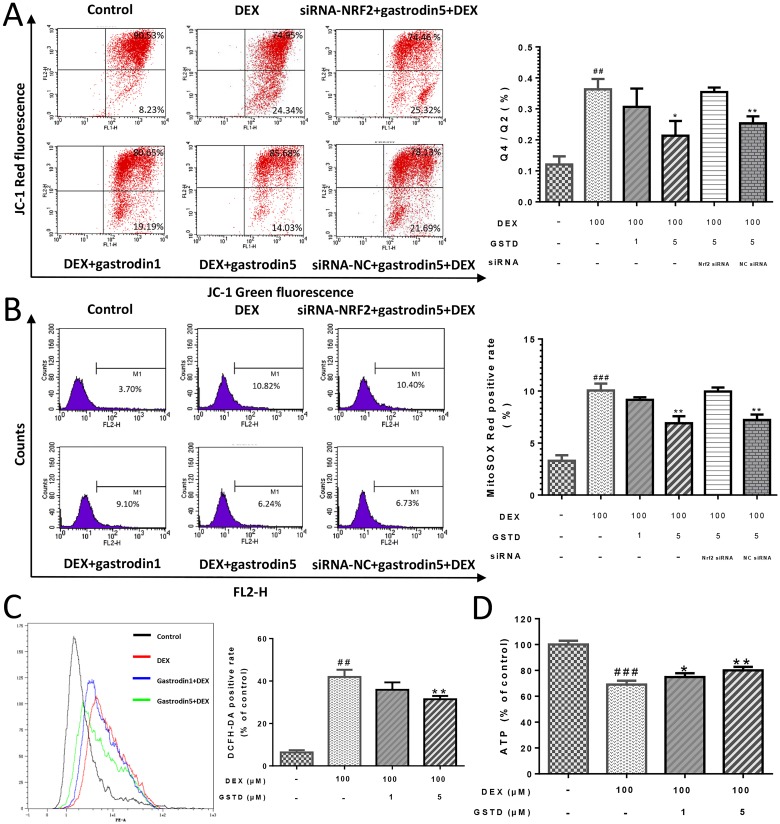
GSTD improved MOMP in DEX induced injury of mitochondria via Nrf2 pathway in primary osteoblasts, performed following JC-1 double fluorescent dye staining (**A**) Cells were pretreated with GSTD (1, 5 μM) for 2 h prior to incubation with DEX (100 μM) for 24 h, followed by flow cytometry detection. GSTD alleviated DEX induced mitochondrial oxidative stress via Nrf2 pathway by cytometry of mitoSOX™ Red staining (**B**). GSTD relieved cellular oxidative stress by cytometry of DCFH-DA staining (**C**) and retrieved ATP production (**D**) as well. ^###^*P* < 0.01 vs control; ^##^*P* < 0.01 vs control; ^**^P < 0.01; ^*^P < 0.05 vs DEX100 (*n* = 3).

A decrease in the red/green fluorescence ratio is manifested as mitochondrial depolarization, which reflects loss of MMP. As shown in Figure 2A, GSTD elevated MMP against DEX treatment in a dose-dependent manner. Knockdown of Nrf2 impaired the maintenance of MMP by GSTD, declaring that this protective effect was established by activating Nrf2.

### Antagonism of DEX-induced mitochondrial and cellular ROS generation by GSTD in primary osteoblasts

Intracellular ROS generation was monitored to investigate whether GSTD could prevent DEX-induced ROS generation. A flow cytometric analysis indicated that the intensity of the fluorescence probe DCFH-DA liberatedsignal increased significantly from DEX exposed cells. However, the peak was markedly shifted to the left in the presence of 1 and 5 μM GSTD (Figure 2C), as were the results of the MitoSOX™ Red test, a mitochondrial superoxide indicator and novel fluorogenic dye that is highly selective for detecting superoxide in mitochondria [16] (Figure 2B). GSTD scavenged DEX-induced mitochondrial and cellular ROS accumulation. As shown in Figure 2B and 2C, DEX increased both mitochondrial ROS production and total cellular ROS level and a portion of the stress-positive cells were upregulated. Hence, ROS decreased in the presence of GSTD in a dose-dependent manner. Besides, GSTD was able to protect mitochondrial function and retrieve cellular adenosine triphosphate (ATP) production, shown in Figure 2D.

### GSTD protects primary osteoblasts by anti-apoptosis

**Figure 3:**
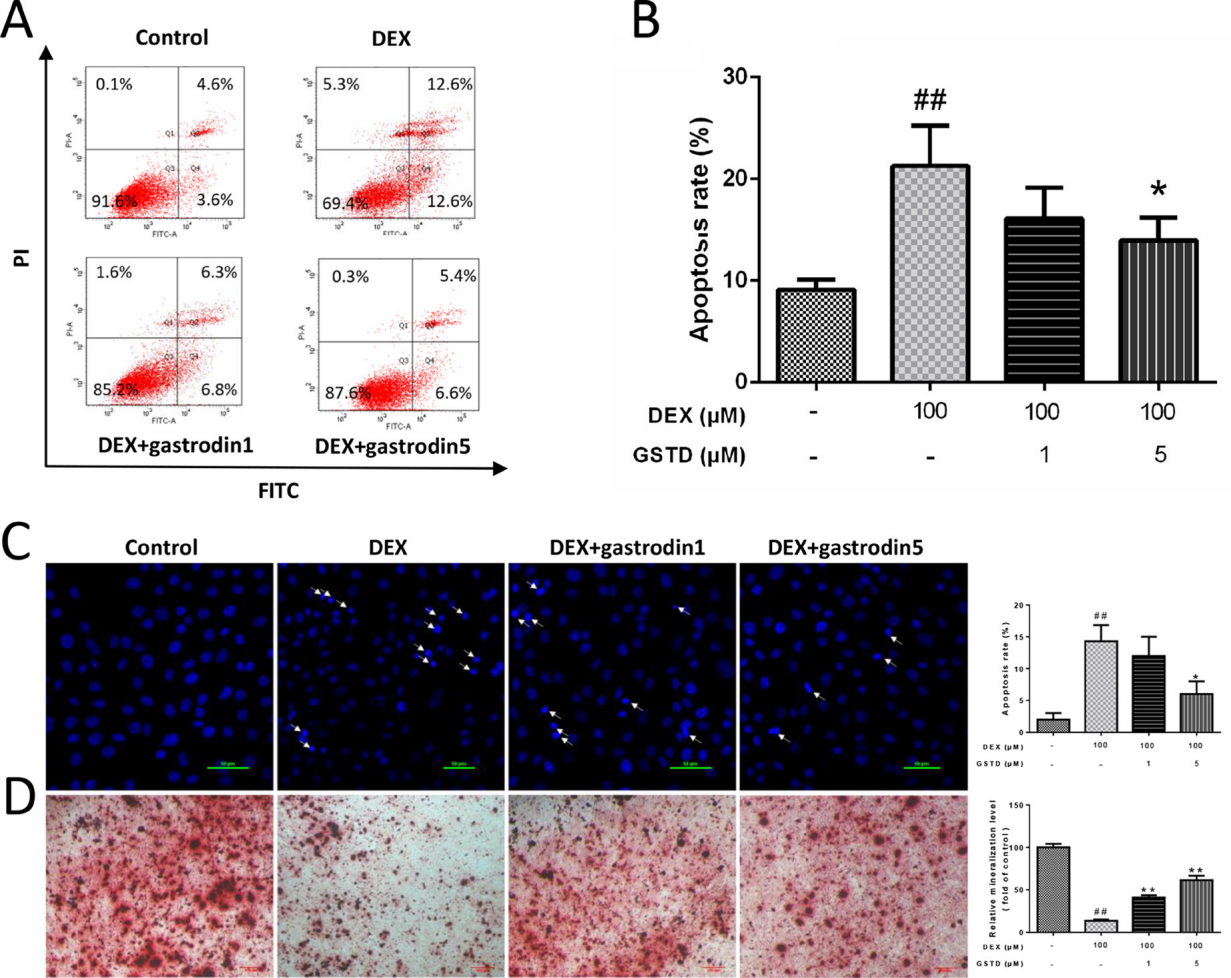
Effects of GSTD on DEX induced apoptosis and differentiation in primary osteoblasts (*n* = 3) (**A**) Cells were treated with GSTD (1, 5 μM) for 2 h prior to incubation with DEX (100 μM) for 24 h. Flow cytometric analysis was used to measure apoptotic rate. (**B**) Levels of apoptotic rate were statistically quantitated shown in column. Data are presented as the mean ± SD. (**C**) Apoptotic cells (arrow) were detected by Hoechst staining. (**D**) GSTD antagonized DEX on promoting osteogenic differentiation and mineralization by alizarin red staining. ^##^*P* < 0.01 vs control; ^*^P < 0.05 vs DEX.

A flow cytometric analysis of Annexin V-FITC/propidium iodide (PI) double staining was performed to determine the rate of cell apoptosis (Figure 3A and 3B). DEX treatment markedly induced cellular apoptosis as reported previously [17], which was validated with a concentration of 100 μM DEX. Moreover, the analysis also showed that cell status improved the combined pretreatment with GSTD, which significantly promoted cellular survival against DEX. In addition, Hoechst staining revealed that the percentage of apoptotic cells increased after exposure to DEX compared with the control, and the number of positive cells (arrow) decreased gradually in the presence of GSTD (Figure 3C).

### GSTD modulated DEX-induced matrix mineralization formation

After a 7 day culture, a microscopic analysis indicated that adding DEX significantly inhibited the formation of calcified deposits, while the use of GSTD preserved calcification of primary osteoblasts and bone mineralization under the DEX condition when compared with that in the control group (Figure 3D). In conclusion, GSTD rescued differential mineralizability of the cells in the DEX treatment.

### GSTD relieves mitochondrial and ER stress

**Figure 4:**
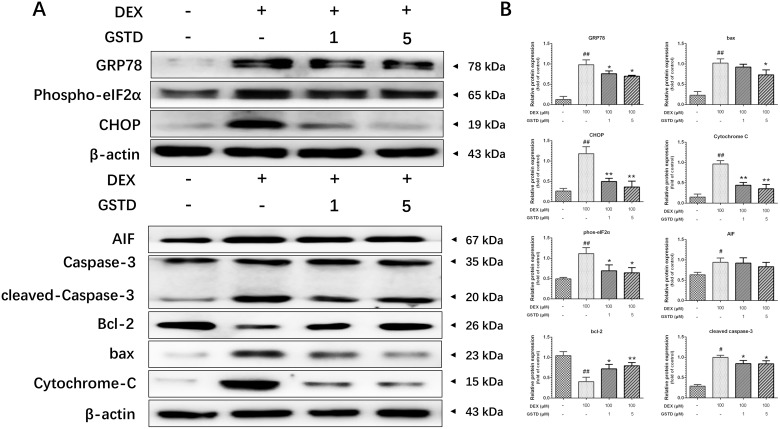
(**A**) Representative Western blots for total protein was analyzed by Western blotting in DEX (100 μM)-induced primary osteoblasts with different-dosed GSTD (1, 5 μM) pretreatment. (**B**) Quantitated levels of protein were statistically quantitated shown in column. The data were expressed as the mean ± SD. ^##^*P* < 0.01, ^#^*P* < 0.05 vs control; ^**^P < 0.01, ^*^P < 0.05 vs DEX (*n* = 3).

Based on the results presented in Figure 4A and 4B, we concluded that DEX had similar functions on ER and mitochondrial stress-related proteins. glucose-regulated protein 78 (GRP78), CCAAT/enhancer-binding protein homologous protein (CHOP), phospho-eukaryotic initiation factor 2 (eIF2), apoptosis-inducing factor (AIF), bax, cytochrome-C and caspase-3, protein levels increased markedly in DEX singly-treated cells, but preconditioning with GSTD lowered the levels of these proteins. Furthermore, GSTD increased the abundance of anti-apoptotic protein bcl-2, blocking apoptotic death.

### The Nrf2 pathway is critical to GSTD and osteoblast survival

**Figure 5:**
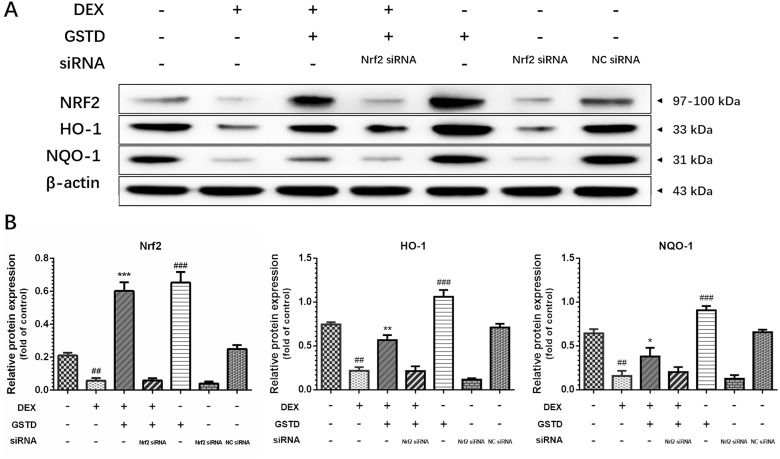
(**A**) Knockdown of Nrf2 by siRNA suppressed GSTD (5 μM)-induced Nrf2 and downstream transcription HO-1 and NQO-1 upregulation under large-dosed DEX incubation. (**B**) Quantitated levels of protein were statistically quantitated shown in column. The data were expressed as the mean ± SD. ^###^*P* < 0.001, ^##^*P* < 0.01, ^#^*P* < 0.05 vs blank group; ^***^P < 0.001, ^**^P < 0.01, ^*^P < 0.05 vs DEX group (*n* = 3).

We further examined the effects of Nrf2 knockdown on cells to explain the instrumental role of Nrf2 and GSTD against DEX-induced osteoblast dysfunction. As displayed in Figure 5, knockdown of Nrf2 by siRNA restrained GSTD-induced Nrf2 upregulation and expression of the downstream transcription factors HO-1 and NQO-1. Similarly, GSTD suppressed DEX-induced MMP loss, which was inhibited by Nrf2 knockdown (Figure 2A). Taken together, these results suggest that Nrf2 is critical for the cytoprotective effects of GSTD against DEX in osteoblasts.

### GSTD improves femoral microstructural and biomechanical properties in the DEX-induced GIO rat model

**Figure 6:**
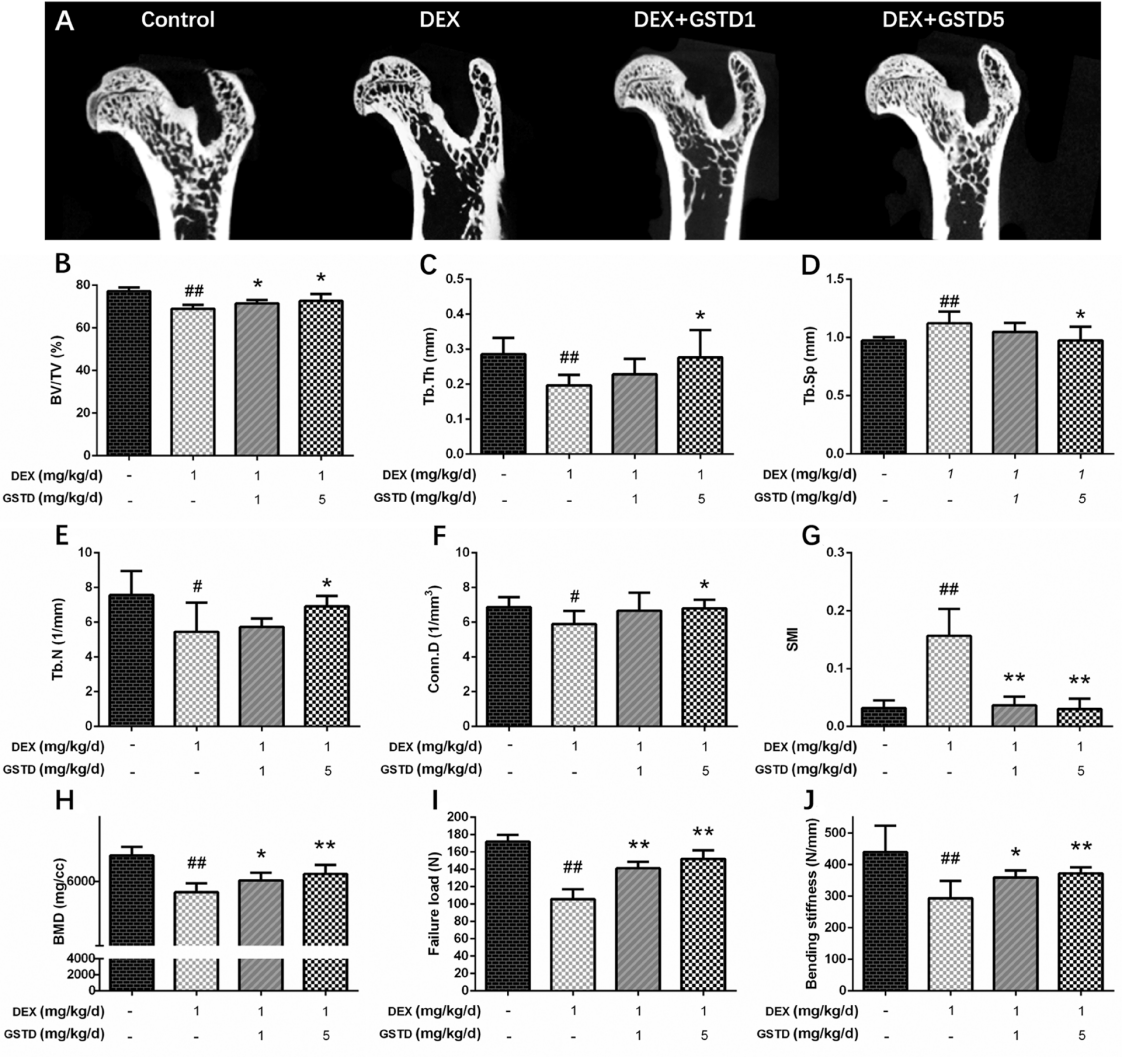
Effects of GSTD on the trabecular micro-architectures in DEX-rats (*n* = 6) Micro-CT analysis within the proximal femur region (**A**). The following computed tomographic indices in the defined region of interest (ROI) were analyzed: BV/TV (**B**), Tb.Th (**C**), Tb.Sp (**D**), Tb.N (**E**), Conn.D (**F**), SMI (**G**). Data from BMD measurements of femurs by DXA (**H**). The failure load (**I**) and bending stiffness (**J**) of femurs were determined by three-point bending test. The data were expressed as the mean ± SD. ^##^*P* < 0.01, ^#^*P* < 0.05 vs control; ^**^P < 0.01, ^*^P < 0.05 vs DEX.

Micro-computed tomography (μ-CT) provided a three-dimensional image of the microstructure of the femur. As shown in Figure 6, data derived from the structural trabecular bone parameters revealed that GSTD upregulated trabecular number (Tb.N; mm^−1^), bone volume/tissue volume (BV/TV;%), trabecular thickness (Tb.Th; mm), and connectivity density (Conn.D), but clearly downregulated trabecular separation (Tb.Sp; mm) and the structural model index (SMI) in femurs (Figure 6A–6G). Bone mass density (BMD) (Figure 6H) also increased, showing the evident improvement of GSTD against erosion of bone microstructure by DEX. Collectively, these data suggest that GSTD improved bone microstructure in a GIO animal model. Failure load (ultimate breaking force, N) on the femoral biomechanical test is the load when breakage occurs and reflects strength of the bone. Displacement at the moment of failure (mm) is called deformation reaching the breakage point. Stiffness to failure (N/mm) was also recorded. Displayed in DEX clearly increased bone fragility and the failure load decreased as well as stiffness compared with the control (Figure 6I and 6J). In contrast, gavage of GSTD somewhat relieved this toxic effect in the DEX-induced osteoporotic rat model.

## DISCUSSION

DEX led to osteoporosis by largely affecting osteoblast function. GSTD, a natural bioactive extract from the traditional Chinese herbal agent Gastrodia elata, is regarded as an Nrf2 activator [13] that effectively removes oxygen free radicals and exerts antioxidant activity in many ways [2]. Based on evidence that DEX could lead to mitochondrial and ER dysfunction resulting in osteoblast apoptosis, the present study focused on organelle-targeted treatment and the full potential of GSTD to relieve DEX-induced osteoblast dysfunction and apoptosis by investigating the possible molecular mechanisms of GSTD on osteoblast survival and differentiation.

Mitochondria have emerged as a crucial mediator of cell survival and apoptosis, as they are involved in the complex metabolic network of cells. Studies have reported that the GCs wear out antioxidant factors or enzymes, indirectly inducing mitochondrial stress. For example, DEX-induced ROS is a harmful by-product of respiration in mitochondria. Mitochondria are the predominant producer of ROS, appearing to be a proapoptotic factor [18] that preferentially directs pro-apoptotic pathways in osteoblasts. Oxidative stress caused by a large dose of DEX results in mitochondrial impairment leading to apoptosis of osteoblasts [19]. How to lower ROS levels in DEX-induced cells and maintain mitochondrial homeostasis to protect cell function remains to be studied. In our experiments, we tested whether GSTD could compete with DEX-induced mitochondrial toxicity. The theory that mitochondria serve as the major intracellular source of ROS has been based largely on experiments with separate mitochondria rather than direct measurements by DCFH-DA at the cellular level. MitoSOX™ Red mitochondrial superoxide indicator is a novel fluorogenic dye that is highly selective for detecting superoxide in the mitochondria of living cells [20]. Loss of the MMP is largely regulated by opening of the mitochondrial permeability transition pores and respiratory chain dysfunction, resulting in damaged mitochondria, even mitochondrial-induced apoptosis, which relies on certain nuclear encoded proteins. Mitochondria are often considered energy powerhouses in cells because they generate most of the ATP, required for metabolism using a specialized electron transport system. GSTD alleviated mitochondrial dysfunction by retrieving cellular ATP level. Mitochondria are not self-supporting entities, and their genome only codes for a few proteins in the electron transport chain, among which the release of pro-apoptotic molecules, such as cytochrome-C [21]. Release of cytochrome-C, an apoptogenic protein, by activated mitochondria into the cytosol triggers caspase proteases that degrade cells via the mitochondrial pathway [3, 22, 23]. Bax is a key regulator of the mitochondrial pathway of apoptosis that accumulates at distinct foci on the mitochondrial surface to undergo a conformational change, oligomerize, and mediate cytochrome-C translocation [24]. AIF induces caspase-independent fragmentation of chromosomal DNA to amplify apoptosis. As our results indicate, DEX activated the caspase3 apoptotic pathway under stressful conditions, increasing pro-apoptotic expression of cytochrome-C and bax, as well as AIF, and initiated the mitochondrial apoptotic pathway. Pretreatment with GSTD blocked this cascade. At the same time, bcl-2 abundance was upregulated, hence inhibiting the progression of osteoblast apoptosis. Osteogenic differentiation is accompanied by formation of mineralized calcium nodules. Alizarin Red staining dye conjugates with the mineralized extracellular matrix as calcified deposits. We observed that GSTD improved osteogenic differentiation against DEX and ameliorated osteoblast mineralization and cellular maturation.

The ER is a sophisticated membrane system and a crucial organelle in cells. It is closely associated with cellular protein synthesis, modification and processing, nascent peptide chain folding, assembly, and transportation. ER stress is a condition in which the homeostasis between protein folding load and ER capacity is disrupted, representing fundamental damage to cell viability and mediating the apoptotic signaling pathway [25]. Large-dose DEX induces the ER stress response in osteoblasts. A large number of studies have indicated that the protein expression levels of GRP78 and CHOP are upregulated accompanied by phosphorylation of eIF2α when severe DEX-induced ER stress occurs in apoptotic osteoblasts [26–28], illustrating that ER stress is strongly implicated in the pathogenesis of GIO. ER stress has captured increasing attention due to its participation in various diseases through ER stress-induced apoptosis [29]. GRP78, also called the immunoglobulin heavy chain binding protein (Bip), is one of the most important ER molecular chaperones that regulates the levels of accumulating unfolded or misfolded proteins. The ER stress response is triggered when these proteins accumulate [30]. CHOP is a primary ER stress pro-apoptotic transcription factor, which has been identified to be the first molecule to prompt ER stress-induced apoptosis [31]. Some evidence indicates that CHOP inhibits expression of bcl-2 and facilitates translocation of the pro-apoptotic protein bax [32, 33]. Our data demonstrate that GSTD lowered CHOP, GRP78, phospho-eIF2α, and bax expression, and promoted the generation of bcl-2, exerting a defensive effect upon DEX-induced ER stress in osteoblasts. ER stress is also an inducer of increased ROS, resulting from the accumulation of misfolded/unfolded proteins, thereby triggering activation of caspase3 apoptosis [5]. GSTD inhibited apoptosis by suppressing activation of cleaved caspase3.

There has been recent interest in Nrf2 in the cell biology field. The Nrf2 signaling pathway is well armed to counterbalance ROS production and is critical for maintaining redox balance in cells [34]. The Nrf2 signaling pathway has also emerged as a regulator of cytoprotection by adjusting mitochondrial function [11]. Several downstream target genes of Nrf2, such as NQO-1 and HO-1, also participate in resisting mitochondrial ROS [35, 36]. Many early studies reported that mitochondrial dysfunction suppresses the function of Nrf2 [37, 38], and an Nrf2 deficiency leads to impaired mitochondria. Activation of Nrf2 has been verified to be beneficial to mitochondria-related disorders [39, 40]. Small molecule activators of Nrf2 help maintain mitochondrial integrity by promoting mitophagy and conferring resistance to ROS-mediated transitions in permeability and improving cellular survivability. Investigators from our laboratory aim to utilize GSTD as a stimulator to ameliorate DEX-ROS-induced mitochondrial dysfunction by stimulating Nrf2 signaling transduction. GSTD was used to induce Nrf2 to withstand the redox-regulated permeability transition and stabilize MMP, suggesting a further role for the Nrf2 pathway in maintaining mitochondrial functionality in osteoblasts. The protective effect of GSTD was weakened, MMP was affected, and downstream NQO-1 and HO-1 decreased as well after knockdown of Nrf2 with transfected siRNA-Nrf2, indicating that GSTD relieved mitochondrial stress by regulating the Nrf2 pathway.

In addition, we designed an *in vivo* experiment to explore the effect of GSTD on bone of the GIO rat model. μ-CT was applied to quantitatively represent the microarchitecture of bone geometry through a range of computed attenuation-based parameters for both *in vivo* and *ex vivo* applications [41]. Additionally, as BMD is responsible for 50%–70% of total bone strength, densitometry was employed as an imperative method to reflect bone quality. The three-point bending test was utilized to determine the bone biomechanical properties. Based on the test results, microstructural parameters [42], such as BV/TV, Tb.Th, Tb.N, Tb.Sp, Conn.D, SMI, and BMD improved with use of GSTD compared to long-term large-dose DEX treatment. Moreover, ultimate breaking force and bone stiffness increased, reflecting the enhancement by GSTD compared to the control and DEX. These results reveal that GSTD could improve bone microstructure and biomechanical strength in the GIO rat model.

Natural extracts from Chinese medicines have been highly esteemed by researchers in terms of their minimal side effects, convenient use, and other advantages. Among all, GSTD, known for its sound medical value, has long been favored by people.This article mainly discussed the pharmaceutical value of GSTD from the perspective of its ability to alleviate DEX-induced mitochondrial dysfunction and ER stress in primary osteoblasts by regulating the Nrf2 pathway and improving bone microstructure and bone biomechanical properties in the GIO model. It might be plausible to expect GSTD to be a promising therapeutic agent targeting DEX-induced mitochondrial and ER stress, which may provide a new approach for preventing bone loss associated with these clinical conditions.

## MATERIALS AND METHODS

### Reagents

Purified GSTD (> 98%) was purchased from the National Institute for the Control Pharmaceutical and Biological Products (Dalian, China). GSTD was dissolved in dimethyl sulfoxide (DMSO; SigmaAldrich, St. Louis, MO, USA) and stored at −20°C. DEX was purchased from SigmaAldrich. The final concentrations of GSTD were 0 (control), 1, and 5 μM, and the final concentration of DMSO in the culture was < 0.01%. Dulbecco Minimum Essential Medium (high glucose) (DMEM) and trypsinEDTA were obtained from GE Healthcare Life Sciences (Hyclone; Logan, UT, USA). The Cell Counting Kit-8 (CCK-8) was purchased from SigmaAldrich. The Reactive Oxygen Species Assay Kit, Hoechst 33258 staining kit, and Annexin V-FITC Apoptosis Detection Kit were purchased from Beyotime Institute of Biotechnology (Jiangsu, China). MitoSOX™ Red mitochondrial superoxide indicator was from SigmaAldrich (St. Louis, MO, USA). Rabbit anticaspase3/cleaved caspase3 (WL02117) was from Wanleibio incorporation, rabbit anti-phospho-eIF2α (cat. no. ab32157), rabbit anti-CHOP (cst. no. 5554), rabbit anti-GRP78 (cst. no. 3177), rabbit anti-AIF (cat. no. ab32516), rabbit anti-Cytochrome C (cat. no. ab13575), mouse anti-bcl-2 (cst. no.15071), rabbit anti-bax (cat. no. ab32503), rabbit antiHO-1 (cat. no. ab68477), rabbit antiNQO-1 (cat. no. ab80588), rabbit antiNrf2 (cst. no. 12721) and mouse antiβ-actin (cat. no. ab8226) monoclonal antibodies were purchased from Cell Signaling Technology Incorporation and Abcam (Cambridge, MA, USA). Primers were designed and synthesized by Sangon Biotech Co., Ltd. (Shanghai, China).

### Animals and groups

Twenty-four 8-week-old female experimental SD rats (weighing 250 ± 24 g) were obtained from the Animal Center of China Medical University. The rats were acclimated to specific pathogen-free laboratory conditions (ventilated controlled room at 20°C on a 12 h light/dark cycle with free access to water and food) for 1 week prior to the drug treatments. The rats were separated into four groups randomly: control, DEX, and DEX with GSTD (1 and 5 mg/kg /day) groups respectively. The GIO rat model (*n* = 6) was induced by an intramuscular injection with 1 mg/kg/day DEX for 8 consecutive weeks, while the control group (*n* = 6) received an equivalent volume of normal saline. The GSTD therapeutic groups (*n* = 12) received different doses of GSTD (1 or 5 mg/kg/day) supplemented by gavage simultaneously after inducing the model with DEX. After 60 days, the rats were euthanized and bilateral femurs and tibias were removed for further analysis. All animal care and experimental procedures were approved by the Institutional Animal Care Ethics and Use Committee of China Medical University, and all efforts were made to minimize the animals’ suffering in accordance with the guidelines.

### Primary osteoblast isolation and culture

Primary osteoblasts were isolated from neonatal rats as described previously [42]. The calvaria were removed from the rats and moderately digested with 0.25% trypsin at 37°C for 30 min. Then, the supernatant was discarded and the remaining bone tissue was continually digested using type II collagenase for 60 min. After centrifugation, the cells were resuspended and maintained in high glucose DMEM supplemented with a 20% fetal bovine serum (PAN-Biotech, Adenbach, Germany), 100 U/ml penicillin, and 100 μg/mL streptomycin at 37°C with 5% CO_2_ in a humidified atmosphere.

### Cell viability after DEX and GSTD treatments

The CCK-8 assay was carried out to detect cell proliferation. Briefly, osteoblasts were seeded at a density of 5 × 10^3^ cells/well in 96-well plates and incubated in growth media for 12 h at 37°C. Subsequently, the cells were exposed to different concentrations of DEX (0, 1, 5, 25, 50, 100, and 200 μM) and GSTD (0, 0.1, 1, 5, 10, and 50 μM) for 24 h. Moreover, another group was created with cells pretreated with GSTD (1 or 5 μM) for 2 h, and then exposed to DEX (100 μM) for 24 h. Subsequently, 10 μl CCK-8 was added to each well and incubated for 2 h. Absorbance was recorded at 450 nm on a microplate reader.

### Alkaline phosphatase (ALP) activity assay

Cells were seeded into 6well plates at a density of 1 × 10^5^ cells/well in different conditioned media. After 7 days of culture, the cells were lysed in 100 μl assay lysis buffer and ALP activity levels (U/ml) were detected with an ALP reagent kit (Nanjing Jiancheng Bioengineering Research Institute, Nanjing, China) according to the manufacturer’s instructions.

### Measurement of mitochondrial membrane potential

The lipophilic cationic fluorescent dye 5, 5′, 6, 6′-terachloro-1, 1′, 3, 3′-tetraethylbenzimidazol-carbocyanine iodide (JC-1) was used to detect changes in the MMP. Cells were cultured in 6-well plates. Measurements were performed using the Mitochondrial Membrane Potential Detection Kit (Beyotime Institute of Biotechnology), according to the manufacturer’s instructions. In brief, cells were collected and incubated with 10 μg/ml of the MMP-sensitive fluorescent dye JC-1 at 37°C for 20 min, washed twice in PBS, and subjected to flow cytometry (Becton Dickinson, Franklin Lakes, NJ, USA).

### Intracellular ATP production measurement

An ATP assay kit (Beyotime, China) was used to detect ATP production. Briefly, whole-cell extracts from indicated cells were lysed by somatic cellular ATP releasing reagent. After mixed with ATP luciferase detection solution, the bioluminescence was recorded on a microplate reader. The protein concentration was measured by BCA protein assay. Emitted light was linearly related to ATP concentration and measured on a microplate reader. Data were normalized to the control group and expressed as percentage of control levels.

### Measurement of mitochondrial superoxide

Mitochondrial superoxide was assayed with the MitoSOX™ Red mitochondrial superoxide indicator (Invitrogen Molecular Probes, Carlsbad, CA, USA). The cells were incubated with 5 μM MitoSOX™ Red at 37°C for 20 min according to the manufacturer’s instructions. After washing the cells with warm buffer, the MitoSOX™ Red fluorescence was detected by FACScan flow cytometry (Becton Dickinson, Franklin Lakes, NJ, USA).

### Flow cytometric analysis of ROS production

Overall intracellular ROS were measured with the nonfluorescent 2,7-dichlorofluorescin diacetate (DCFH-DA) assay. After the treated cells were harvested and rinsed in PBS, they were incubated in 10 μM of the DCFH-DA florescent probe at 37°C for 30 min. The samples were analyzed by FACScan flow cytometry (MACSQuant Analyzer 10, Miltenyi Biotec, Gladbach, Germany).

### Flow cytometric analysis of osteoblast apoptosis

Osteoblasts were incubated in 6-well plates at a density of 2 × 10^5^ cells/well for 24 h. Then, the cells were exposed to DEX in the presence or absence of different concentrations of GSTD for the indicated time periods. The cells were harvested, resuspended in 500 μl binding buffer containing 5 μl Annexin V-FITC and 5 μl PI according to the manufacturer’s instructions, rinsed twice with PBS, and placed in an ice bath for 30 min. All samples were analyzed by FACScan flow cytometry (Becton Dickinson, Franklin Lakes, NJ, USA).

### Hoechst staining assay

Osteoblasts were inoculated on coverslips in 6-well plates at a density of 8 × 10^4^ cells/well. After reaching 80% confluence, the cells were exposed to the corresponding treatment for the corresponding time. The cells were fixed for 20 min and rinsed twice with PBS. The cells were incubated in 10 μg/ml Hoechst 33258 working solution at room temperature for 5 min. After washing with PBS, the coverslips were mounted inversely onto slides with an anti-fluorescein quencher and observed under a laser scanning confocal microscope (C2 plus; Nikon, Tokyo, Japan) under blue light with an excitation wavelength of 350 nm.

### Mineralization and alizarin red staining

Primary osteoblasts were cultured on 6-well plates in osteogenic inducing medium that was replenished every 2 days. After 7 days of culture, the cells were fixed in 90% ethanol at room temperature for 30 min. After rinsing with distilled water, the cells were stained with 1 ml of 40 mM Alizarin Red-S (pH = 4.2) per dish at room temperature for 20 min with gentle shaking. After aspirating the unincorporated dye, the cells were washed three times with 2 ml distilled water to exclude non-specific staining. Mineralized nodules and stained cells were visualized and photographed by microscope (Carl Zeiss Inc., Oberkochen, Germany).

### Transient transfection with Nrf2 siRNA

Silencing of Nrf2 in primary osteoblasts was carried out by using a small interfering ribonucleic acid (siRNA) assay kit (GenePharma Technology, Shanghai, China). (sense 5′-CGAGAAGUGUUUGACUUUATT-3′, antisense 5′-UAAAGUCAAACACUUCUCGTT-3′) was used for Nrf2 knockdown. Briefly, osteoblasts were transfected at 70%–80% confluence with the siRNA–Lipofectamine 3000 complex (Invitrogen). The culture medium was replaced with DMEM (high glucose) medium after 5 h.

### Western blot analysis

Total proteins from each well were harvested in ice-cold radioimmunoprecipitation lysis buffer (Thermo Fisher Scientific, Inc., Waltham, MA, USA) supplemented with phenylmethanesulfonyl fluoride for 1 h. The protein concentration was assessed with a bicinchoninic acid protein assay kit (Beyotime Institute of Biotechnology, Haimen, China), according to the manufacturer’s instructions. Equal protein content from each treatment was separated by 12% sodium dodecyl sulfate polyacrylamide gel electrophoresis and electrophoretically transferred onto polyvinylidene difluoride membranes (Millipore, Bedford, MA, USA). The membranes were soaked in 5% skimmed milk as a blocking buffer for 1 h, then washed in Trisbuffered saline Tween20 three times at room temperature. The membranes were incubated with primary monoclonal antibodies against GRP78, CHOP, phospho-eIF2α, AIF, bcl-2, bax, cytochrome-C, Nrf2, NQO-1, HO-1, and caspase3/cleaved-caspase3 overnight at 4°C followed by hybridization with horseradish peroxidase-conjugated secondary antibody. Relative protein levels were calculated based on β-actin as the loading control. Signal detection was visualized by enhanced chemiluminescence.

### Micro-computed tomography

μ-CT was conducted on the proximal left femur, utilizing a micro-CT system (QuantumGX, PerkinElmer, Hopkinton, MA, USA) to scan a three-dimensional image and microstructure of the femur as well as precisely analyze the same region of interest. The specimen scanner settings were recommended by the manufacturer; exposure time was 14 sec at 90 kV and 88 μA with a resolution of 2 μm, and field-of-view of 12.8 × 12.8 mm. The structural parameters for trabecular bone were derived from μCT data, including Tb.N; mm^−1^, BV/TV;%, Tb.Sp; mm, Tb.Th; mm, Conn.D, and the SMI, which were evaluated based on traditional static bone histomorphometry.

### Bone mass densitometry

Densitometry was performed on right femurs by dual energy X-ray absorptiometry using a PIXImus II densitometer (GE Medical Systems, Lunar Division, Madison, WI, USA), and data were recorded for further analysis. The measurement was limited to the proximal femur areas of the rats.

### Biomechanical analysis

Three-point bending was performed on the right femurs to determine the bending properties of the cortical bone. After euthanasia, soft tissue attached to the bone was eliminated. Each right femur was removed as a test specimen. A microcomputer-controlled electronic universal testing machine (Instron 4202; Instron, Canton, MA, USA) equipped with an 18 mm gauge length was operated. The femurs were positioned on two lower supporting points on the anvil. A load was applied to the middle of the femur with a displacement speed of 1 mm/min for all tests until the femurs broke. During the biomechanical test, failure load and failure displacement were defined as the change in the value until occurrence of the overt fracture for three-point bending. Finally, failure load (N) and failure displacement (mm) were recorded, and stiffness (N/mm) was calculated.

### Statistical analysis

All the presented data and results were confirmed using GraphPad Prism 6.01 and were expressed as mean ± SD in at least three independent experiments. One-way analysis of variance was used to calculate the statistical variance. *P* < 0.05 (^*^), *P* < 0.01 (^**^) or *P* < 0.05 (^#^), *P* < 0.01 (^##^) level was considered to indicate statistically significant difference.

## Abbreviations

(GCs): Glucocorticoids
(GIO): glucocorticoid induced osteoporosis
(GSTD): gastrodin
(DEX): dexamethasone
(MMP): mitochondrial membrane potential
(AIF): bax and apoptosis inducing factor
(ER): endoplasmic reticulum
(ROS): reactive oxygen species
(Nrf2): nuclear factor erythroid derived 2-related factor-2
(NQO-1): NAD (P) H: quinone oxidoreductase 1
(HO-1): heme oxygenase 1
(CCK-8): Cell Counting Kit-8
(siRNA): small interfering ribonucleic acid
(Tb.N): trabecular number
(BV/TV): bone volume/tissue volume
(Tb.Sp): trabecular separation
(Tb.Th): and trabecular thickness
(Conn.D): connectivity density
(SMI): structure model index
(BMD): Bone mass density
(GRP78): glucose-regulated protein 78
(CHOP): CCAAT/enhancer-binding protein homologous protein
